# Promoting self-change in cannabis use disorder: Findings from a randomized trial

**DOI:** 10.3389/fpsyt.2022.1015443

**Published:** 2022-11-22

**Authors:** Magdalen G. Schluter, David C. Hodgins, Jonathan N. Stea, Michelle L. Kilborn

**Affiliations:** ^1^Addictive Behaviours Laboratory, Department of Psychology, University of Calgary, Calgary, AB, Canada; ^2^Department of Psychology, University of Calgary, Calgary, AB, Canada; ^3^Werklund School of Education, University of Calgary, Calgary, AB, Canada

**Keywords:** cannabis, marijuana, cognitive-behavioral therapy, motivational interviewing, self-directed intervention, treatment

## Abstract

**Background:**

A growing body of literature supports the efficacy of cognitive-behavioral therapy (CBT) and motivational interviewing (MI) for the treatment of problematic cannabis use, diagnostically referred to as cannabis use disorder, though most individuals do not access formal treatment. Stepped-care-type models emphasize interventions across a continuum of severity and there is a need for more treatment options across this continuum. This project focused on the evaluation of the least intensive of the individual interventions – promotion of self-directed recovery.

**Methods:**

Using a three-arm randomized control trial design, adults (*N* = 186) with problematic cannabis use and who wished to recover with minimal professional support were recruited from across Canada and randomized to receive a self-directed treatment workbook based on CBT and MI principles (WB; *n* = 61), the workbook plus a single MI session (WMI; *n* = 61) or a delayed treatment control (DT; *n* = 65) condition. Participants completed 3-month and 6-month follow-up assessments.

**Results:**

Across conditions, GEE modeling revealed that the baseline to 3-month slopes differed significantly from zero, *ps* < 0.001. Participants in the WMI condition reduced their frequency of use to a greater extent than the WB alone, *p* = 0.005, and DT groups, *p* = 0.02. Chi-square analysis revealed that participants in the WMI condition also showed greater rates of abstinence at 3-months follow-up than participants in the WB or DT condition, *p* = 0.046. Changes in the frequency of cannabis use between 3-months and 6-months did not differ significantly between groups, *p*s > 0.05. For quantity of cannabis use, a significant effect of time emerged, *p* = 0.002. However, no between-group effects were significant from baseline to 3-months, or from 3- to 6-months, *p*s > 0.06.

**Conclusion:**

Overall, results support the utility of a brief self-directed workbook in combination with a single MI session at promoting changes in cannabis use. This self-directed intervention has the potential to fill an important need in that the self-directed intervention can attract individuals who wish to recover with minimal professional support.

**Clinical trial registration:**

[https://www.isrctn.com/], identifier [ISRCTN426 32893].

## Introduction

In 2018, the Canadian federal Cannabis Act legalized recreational cannabis distribution and use, with a key objective of protecting public health (1). The ease of accessibility of cannabis and normalization of its use due to legalization have raised significant concerns regarding potential increases in heavy use and, consequentially, problematic cannabis use and its associated harms (2). Problematic use is diagnostically referred to as cannabis use disorder (CUD) and ranges from mild to moderate to severe (3). A successful public health approach to non-medical cannabis use, in addition to prevention and consumer protection, must also include a range of intervention options for individuals with problematic cannabis use (4). Stepped-care-type models contribute effectively to a public health approach. They emphasize interventions across a continuum of severity, ranging from public awareness messages to encourage responsible use and readiness to change to intensive inpatient or outpatient treatment services for individuals with severe problems (5, 6). This project focuses on evaluation of the least intensive of the individual interventions - promotion of self-recovery, commonly referred to as self-directed change.

Paradoxically, despite low rates of treatment-seeking among people with CUD, the demand for treatment is increasing across the globe. Cannabis is the most frequent psychoactive substance reported by treatment-seekers in North America, Central and South America, Africa, European Union countries, and Australia (7). This is likely due, in part, to increases in the frequency of use, the number of people with cannabis use disorder, and increased awareness of problems associated with cannabis use. The potency of cannabis products has also increased dramatically in the past decade (8), which could arguably contribute to the increased rates of CUD, although this relationship has not yet been established.

Fortunately, a growing body of literature supports the efficacy of several psychological interventions for cannabis problems. The utility of two complementary treatment models, cognitive-behavioral therapy (CBT) and motivational enhancement therapy [MET; (9–11)] have been investigated and shown to be efficacious for the treatment of CUD by groups of independent researchers (9–14). CBT models help clients to understand the contingencies of substance misuse, and to develop relapse prevention and coping skills (15). Common techniques include learning what situations, people, and objects can trigger cravings or a desire to use, increasing awareness of thinking patterns that contribute to continued cannabis use, and identifying high-risk situations. MET is based on Motivational Interviewing (MI) principles and seeks to enhance motivation to change substance use behavior by providing non-judgmental feedback, resolving ambivalence, and via goal setting (15).

Evidence suggests that a combination of CBT and MET is most efficacious for CUD (9, 16, 17). The Marijuana Treatment Project [MTP; (18)] examined the efficacy of a CBT/MET treatment across three demographically diverse treatment sites; adults with CUD were randomized to receive a 2-session MET intervention, a 9-session MET plus CBT and case management intervention, or a delayed treatment control. At 15-months follow-up, individuals in both active interventions showed greater reductions in cannabis use and problems relative to the control condition. Additionally, individuals in the MET plus CBT and case management intervention demonstrated the greatest reductions in the frequency of cannabis use and symptoms of addiction (18). Similarly, the CANDIS treatment program (19, 20) examined the efficacy of a 10 session CBT/MET plus problem solving treatment program in Germany. Adults with CUD who received 10 sessions of CBT/MET plus problem solving showed greater rates of abstinence, reduced frequency of cannabis use, and reduced cannabis-related problems compared to adults in the waitlist control. Most of these gains were maintained at six-months (19). The Cannabis Youth Treatment [CTY; (11)] study also found a 5-session MET plus CBT intervention to be as effective as other more intensive and costly treatments. In sum, MET plus CBT is an effective treatment for CUD among treatment-seeking individuals.

Although the literature supports the efficacy of CBT + MET in the treatment of CUD, relatively few individuals will seek available treatments. The most frequently cited reasons that many individuals are not willing to seek treatment are embarrassment (i.e., stigma) and a desire to “do it on their own” (21, 22). This latter reason supports the finding that the most common pathway to recovery for CUD and for other addictions is recovery without treatment (23). A stepped-care approach may enhance the provision of treatment for CUD by providing individuals with the opportunity to choose a level of intervention that is consistent with their goals and preferences, such as self-directed change. Self-directed interventions also overcome many of the perceived limitations evident in formal treatment (i.e., availability, level of intensity). They support the desire to recover with minimal support and are also relatively inexpensive, accessible, and have reduced stigma compared to formal treatment. In this context, formal treatment refers to psychosocial treatment with a mental health professional, in an individual or group context or attendance at a mutual support group (such as a 12-step group). Several lines of research suggest that augmenting natural recovery with cognitive-behavioral and motivational tools can promote recovery in a larger population than is reached by formal treatment, and it may be preferable to many individuals. First, formal treatment is a limited resource, and such interventions are typically of interest to people with more severe problems. However, addiction severity falls on a continuum from mild to moderate to severe (3), and individuals with mild to moderate problems comprise a significant proportion of individuals with addictive disorders, including CUD (24). These individuals are also in need of support. Although many individuals with CUD will initiate a self-change process, they tend to have five to six years of problematic use before this occurs (21, 25). Moreover, few of these individuals with CUD will seek available treatment.

Second, brief interventions that facilitate self-change have demonstrated effectiveness with other addictive disorders (24, 26). These brief, self-directed interventions typically utilize self-directed written materials, worksheets, and provision of personalized feedback (24). For example, in our lab, we have developed a self-recovery program for problem gambling that involves a self-directed workbook with a motivational interview conducted via telephone (27–30). This treatment has been recognized as an evidence-based intervention by the US National Registry of Evidence-based Programs and has been adapted for use in a variety of countries (31) and seven languages, illustrating that it can be scaled for national accessibility. Several self-directed treatments have been developed for cannabis use in adults, though they have been largely limited to web-based approaches (32–34). One study by Rooke and colleagues (32) tested *Reduce Your Use*, a self-directed online treatment program among a sample of 225 individuals seeking to reduce or stop using cannabis. Participants were assigned to either the treatment program which consisted of modules based on cognitive, motivational, and behavioral principles or assigned to a cannabis information control condition. The intervention group showed significantly lower frequency of cannabis use at 3-months follow-up, but not lower quantity. In contrast, Sinadinovic and colleagues (33) found no benefit of an online treatment program with optional therapist communication via chat compared to a waitlist control group. Nevertheless, a recent meta-analysis of nine web-based interventions for prevention and treatment of CUD highlighted the potential utility of such interventions (35). As such, self-directed treatments for CUD appear promising. However, workbook-based treatments that have demonstrated utility with other addictive disorders have not been considered in the context of cannabis.

Third, research in our lab has demonstrated that recovery from CUD without treatment is common, and that individuals who recover without treatment experience (i.e., natural recovery) show similar change-processes to those who experience treatment-assisted recovery (25). In one study, we recruited individuals who had recovered from CUD (*N* = 119) with formal treatment or via natural recovery (25). Both groups showed remarkably similar motivators and processes of recovery. Individuals in both groups provided the same most cited motivations for reducing problematic cannabis use; Namely they reported that their use became inconsistent with their self-image and lifestyle, and that it led to perceived psychological problems. These results are consistent with other studies that have previously reported motivations in individuals who had sustained only short-term treatment goals at the time of the study (36–38), lending confidence to our findings. Additionally, individuals in both groups described utilizing the same cognitive strategies (e.g., considering the positive and negative consequences of cannabis use) and behavioral strategies (e.g., avoidance of high-risk situations) as part of the recovery process.

A second report that examined individual experiences to gain a richer understanding of the recovery process, showed that both groups most often attributed their recovery success to cognitive and motivational factors, consistent with the previous analyses (39). This pattern of change processes in CUD has also been demonstrated with other addictive disorders such as alcohol, other drugs, and gambling, and has fueled the development of brief interventions that facilitate self-change (24, 26). Most participants in both groups reported that they would recommend both formal treatment and self-help materials to another person experiencing concerns related to their cannabis use. However, treatment-assisted participants who had chosen moderation goals (i.e., to moderate their use versus quitting) were more likely to recommend natural recovery compared to those who had chosen abstinence goals. Given that most treatment programs emphasize abstinence (9), rather than also supporting moderated use, there may be a lack of fit between personal moderated use goals and the abstinence goal imposed by treatment programs. This lack of fit may partly explain why treatment-assisted participants who had chosen moderation goals were less likely to recommend treatment-assisted recovery. Taken together, these findings highlight the perspective of individuals with CUD recovery experience, which is critical to planning effective interventions that individuals are likely to utilize (40).

In sum, research suggests that a hybrid approach of two complementary therapeutic models, CBT and MET is an effective treatment for CUD. However, many individuals are unable to receive formal treatment, due to limited availability, or are unwilling to seek treatment because of stigma or a desire for natural recovery. Fortunately, brief interventions that facilitate self-change are effective, and similar change processes are observed in both treatment-assisted and natural recovery. These change processes may be utilized to provide support to individuals through a brief self-directed intervention. An intervention that can attract individuals who wish to recover with minimal professional support would also bridge the current mismatch between current treatment needs and available services and fill an important role within an integrated public health approach.

The aim of the present research was to test the clinical utility of a brief self-directed intervention for individuals with problematic cannabis use who wished to recover with minimal professional support. The main objectives were to determine whether a self-directed workbook package could produce significant change in cannabis use among individuals with problems associated with cannabis use in the short-term (up to 6-months), and the benefit of brief motivational interviewing in combination with the self-directed treatment. To this end, a three-arm randomized control trial (RCT) design was utilized, and participants were randomly assigned to (i) receive the self-directed workbook alone (WB); (ii) receive a brief motivational interview in addition to the workbook (WMI); or to a delayed workbook treatment control condition (DT). We had three primary *a priori* hypotheses:

(1). Participants in the WMI and WB groups would show significantly lower frequency and quantity of cannabis use at 3-months follow-up than those assigned to the DT group, as individuals in the DT group would have not yet received the workbook.

(2). Participants in the WMI and WB groups would show greater significantly greater rate of change in their frequency and quantity of cannabis use than those in the DT group. This difference was expected to be most pronounced between baseline and 3-months, versus between 3-month and 6-months follow-up. Between 3- and 6-months, it was predicted that the rate of change for participants in the WMI and WB groups would slow, having already made significant gains, whereas participants in the DT group would show an increased rate of change, having received the workbook at 3-months.

(3). Participants who received a motivational interview (WMI condition) would show greater reduction in the frequency and quantity of cannabis use than participants who received the workbook alone (WB condition) or participants in the DT condition.

## Materials and methods

### Study design

The current study utilized a three-arm randomized control trial that compared the efficacy of a self-directed treatment workbook alone and in combination with a brief motivational intervention in its ability to reduce problematic cannabis use and associated problems. The two intervention groups were compared against each other and against a wait list control group in which participants received a baseline assessment and access to the workbook following a three-month waiting period. Participants completed a follow-up assessment three months and six months after the baseline assessment.

Following completion of the baseline assessment, participants were assigned to one of three groups, stratified by gender and probl em severity (CUDIT-R < 22 or > 23): (i) workbook plus motivational interview (WMI); (ii) workbook only (WO); or (iii) delayed workbook treatment control (DT). The *blockrand* package (41) in R version 4.0.3 (42) was used to create stratified random assignments within randomly chosen block sizes of 3, 6, 9, and 12. This procedure allows for relatively equal sample sizes across groups without selection bias (43).

### Recruitment procedures

Adapting earlier procedures (30), online media announcements across various platforms were used to recruit Canadian residents who were concerned about their cannabis use and who were interested in self-directed change. To mitigate risk of participants misreporting symptoms to be eligible for studies with explicit inclusion criteria, a two-stage screening process was utilized (44, 45). Participants were first directed to Qualtrics and asked to complete a brief screening survey. Attempted survey completions from a Virtual Private Server were automatically detected and blocked to ensure that participants completing the survey were in Canada at the time. IP addresses were automatically and manually checked for duplicate response attempts.

Eligibility criteria were adapted from a previous brief intervention for CUD (12) and previous research in our lab on self-change interventions (28): (a) 18 years of age or older; fluent in English; (b) perception of a cannabis use problem; (c) a score of *13* or greater on the Cannabis Use Disorders Identification Test-Revised [CUDIT-R; (46)]; (d) had used cannabis at least once in the past month; and (e) not currently receiving any other treatment for cannabis use problems (including 12-step programs and any medical or psychological treatment where cannabis problems are addressed). Participants were not excluded from the study based on engagement in other potentially addictive substances or behaviors, though this was assessed at baseline.

Interested participants who met the eligibility criteria outlined above were invited to complete the baseline assessment where these criteria were confirmed. Additional eligibility criteria included: (f) consistent responding, characterized by scores on the CUDIT-R that did not differ by more than three points from the score received at screening; and (g) provision of a valid Canadian address where the workbook could be mailed.

#### Sample justification

We aimed to recruit a sample of 120 participants (40 per group). We estimated [based on (28)], that we would successfully follow at least 102 at 3-months and 90 and 6-months. *A priori* power calculations were conducted for a clinical superiority trial with continuous outcome variables. Based upon estimated baseline scores on the primary outcome variable (days of cannabis use), this sample would be able to show statistically significant baseline to follow-up effects with power = 0.90; For days of use in past month, assuming a baseline mean of 25.38 (SD = 6.2; Stephens et al. (14), and a reduction to 17.09 days in the delayed workbook treatment control group, then a sample of 40 per group would be sufficient to reliably show a reduction of 10 days or more in the workbook group. Consistent with Hodgins and colleagues (28), clinical significance in the present study was defined as a reduction in cannabis by at least 50% or sustained abstinence for the preceding 30-days.

### Trial interventions

#### Workbook

These participants received a mailed self-directed workbook. The workbook was developed based on the results of research that identified the most common behavioral and cognitive-motivational strategies used by individuals who have successfully recovered from CUD (25) Table 1]. It includes four core modules (self-assessment, goal setting, meeting your goal, and maintaining your goal). Examples of strategies are understanding the main reasons for using cannabis and reasons for changing (motivational), identifying and managing triggers (cognitive/behavioral), identifying and challenging patterns of thinking that increase risk of use (cognitive), and increasing social supports (behavioral). The workbook also provides information about provincial and territorial resources for further support if the self-directed approach is ineffective.

**TABLE 1:** Contents of the workbook and corresponding strategies.

No.	Content	Identified strategies from Stea et al. (25)
S0	**Introduction**	
	Information about cannabis use disorder, its signs, and cannabis withdrawal	
S1	**Self-assessment**	
	Is there a problem?	
	Understanding your cannabis use	
	Understanding your reasons for using cannabis	
S2	**Making your decision**	
	Understanding reasons for changing your cannabis use	Identifying reasons for resolution
	Pros and cons of cannabis use	Thinking about the negative consequences and the benefits of not using cannabis
	Choosing a change goal	
	Personal commitment to self	Accountability as a maintenance factor
S3	**Reaching your goal**	
	Triggers and cravings	
	*Dealing with urges/cravings*	Hobbies/distracting activities
	*Identifying triggers*	Identifying triggers
	*Managing triggers*	Stimulus control/avoidance
	*Planning ahead*	Identifying high risk situations
	Changing thinking	
	*Identifying self-talk*	
	*Challenging unhelpful thoughts*	Changing patterns of thinking and attitudes
	Increasing social supports	Decreasing time spend with users/increased time spent with non-users and social/family support
	Diet and exercise	Exercise/diet changes
	Focusing on goals and values	Setting and focusing on life goals
S4	**Maintaining your goal**	
	Planning ahead	Coping with stress and triggers
	Peer pressure and refusal skills	Exposure to peer pressure as a reason for relapse
	Slips and relapses	
	Dealing with other life Problems	

#### Workbook plus motivational interview

Participants assigned to the WMI condition received the self-directed workbook following a brief motivational interview conducted over Microsoft Teams with audio only. The motivational therapist contacted the participant as soon as possible to schedule the motivational interview, which were generally conducted within two weeks of the baseline assessment (*M* = 12.82; SD = 6.53).

The motivational telephone interaction was modified from the well-validated manualized MI protocol for gambling disorder. The interview attempted to explore ambivalence and strengthen the participants motivation for changing their cannabis use. The interview began with inviting participants to share their reasons for signing up for the study and reasons for wanting to change their behavior. The motivational interviewing approach is guided by five therapeutic principles (47): acceptance of the individual and recognition that ambivalence is a normal process; development of discrepancies between the individual’s current behavior and their goals or values; avoidance of argumentation; rolling with resistance; and supporting the individual’s self-efficacy. The interviews ended with a brief description of the workbook and interviewers drew a connection between a specific workbook section and the client’s own ideas for change. The interviews were an average 44.95 min in length (SD = 11.15; range 26-82) and were audiotaped.

##### Therapist adherence

MI interviewers (*N* = 6) were graduate students in a Clinical Psychology program who were trained in the MI protocol by a Clinical Psychologist who is experienced with training clinicians in MI. Training involved directed readings in MI, a training workshop, supervised role-plays, and supervision on two initial interviews. Interviewers were required to demonstrate competence in the MI protocol through role-plays. In the two initial interviews, interviewers were assessed in their ability to use 17 required elements in the MI protocol (e.g., addressing physical and emotional concerns; promoting self-efficacy; asking about previous change attempts) and three prohibited elements (providing unsolicited advice, using the “righting reflex,” and confrontation). The range of required elements present in the interviews was 15 to 17 (*M* = 16.40). There were no instances of prohibited elements in the interviews reviewed.

#### Delayed workbook treatment control

These participants were assigned to a 3-month delayed treatment control condition. Participants were informed that they would receive the workbook following a waiting period of 3 months. Following the waiting period, participants invited to complete a follow-up assessment and to provide their address where the workbook could be mailed.

### Baseline assessment

#### Measures

##### Demographic questionnaire

A lab-developed questionnaire recorded age, gender identity, ethnicity, marital status, level of education, and household income.

##### Cannabis use disorders identification test-revised [CUDIT-R]

The CUDIT-R (46) is an eight-item screening measure for problem cannabis use in the past 6-months. Scores of 8 or more indicate risky cannabis use, while scores of 12 or more suggest a possible CUD. It shows good internal reliability and concurrent validity (48), and high sensitivity and specificity for identifying moderate CUD with a threshold of 13 (49). The CUDIT-R was administered in the screening and baseline surveys to stratify the random assignment by CUDIT-R score, as reported in *Recruitment Procedures*. The internal reliability for the present study was α = 0.61.

##### Marijuana problem scale [MPS]

The MPS (13, 14) is a 19-item measure which assesses the impact of use in social, financial, work, physical health, cognition, self-esteem, motivation, and legal domains in the previous month. The number of problems on the MPS is sensitive to change, and can be used to assess changes in use-related problems after treatment (14). Internal reliability for the present study was α = 0.87.

##### Marijuana problem scale lifetime version [MPS-L]

The MPS-L (50) is a 16-item measure of lifetime problems associated with cannabis use. It yields a total score and two sub scores that reflect internal and external consequences. It is adapted from the MPS and shows good internal and test-retest reliability (50). Internal reliability for the present study was α = 0.87.

##### Cannabis engagement assessment [CEA]

The CEA (51) contains 30 questions that assess the quantity, frequency of use, and method of consumption for dried cannabis products (excluding edibles), cannabis concentrates, and edible products. For each method, several indices of cannabis engagement can be calculated, that integrate both frequency and quantity of use (e.g., the overall amount of cannabis product consumed through a given mode). It includes a question that assesses overall frequency of cannabis use in the previous 30-days. The overall quantity of cannabis use estimated across all three modes of cannabis in a single composite variable can also be calculated. Two additional sections assess other factors associated with cannabis use and history of use.

##### Screener for substance and behavioral addictions [SSBA]

The SSBA (52) is a brief screening instrument for self-attributed problems with four substances (alcohol, tobacco, cannabis, and cocaine) and six behaviors (gambling, videogaming, binge eating, shopping, sex, and work) in community samples. Scores range from 0 to 16, with higher scores indicating greater risk of addiction. It was developed from a larger pool of items that were generated by content-coding responses to open-ended questions asking individuals what signs or symptoms they felt were important indicators of problematic engagement (53). Internal consistency for the present study ranged from α = 0.77 (Cannabis) to α = 0.96 (Tobacco).

##### Kessler psychological distress scale [K10]

The K10 (54) is a brief, well validated measure of psychological distress that is sensitive to changes over time (54, 55). The internal reliability for the present study was α = 0.93.

##### World health organization quality of life-8 item scale [WHOQoL-8]

The WHoQoL-8 (56) is an eight-item version of the longer WHOQoL, a self-report measure of quality of life. It has robust psychometric properties, and correlates strongly with the WHOQoL (56). Scores range from 1 (“Very Satisfied”) to 5 (“Very Dissatisfied”). An average score across items is calculated, where higher values indicate lower quality of life. Internal consistency for the present study was α = 0.84.

### Follow-up assessment

Follow-up assessments were conducted at 3-months and 6-months post-baseline with a completion rate of 82.80 and 76.34%, respectively. Follow-up rates did not differ significantly by group, *ps* > 0.94.

At each follow-up assessment, the following measures were re-administered: CEA, MPS, K10, WHOQoL-8, and change goal. Participants also were asked whether they had utilized other forms of treatment in the previous 3-months, how successful they had been at reaching their treatment goal (on a scale of 0-“nothing has changed” to 10-“I reached my goal”), how helpful they found the workbook at helping them work toward their goal (on a scale of 0 – “I could have made as much progress without the workbook” to 10-“the workbook has been very helpful”), and how often they utilized the workbook (0 – “Never” to 5 – “Daily”).

### Data analysis

Analyses were conducted using SPSS version 28.0 except generalized equation modeling (GEE), which was done in R (42) using *geepack* (57). Two primary outcome variables were decided *a priori* to assess the success of the intervention at producing a statistically significant improvement: mean number of days of cannabis use and the overall amount of cannabis used in the previous month. Self-rated improvement, psychological distress, and quality of life were used as secondary outcome variables. A missing values analysis that also included baseline characteristics showed that data were missing completely at random, Little’s MCAR test χ^2^ = 3599.86, *p* = 0.22). Thus, analyses were conducted on the intent-to-treat sample with all available data.

For the 3- and 6-month outcomes, the cannabis use variables were calculated for the 30 days prior to pretreatment and prior to each follow-up assessment. Extreme outliers, identified using the 3*interquartile range method, were recoded as 1 less/greater than the smallest/largest non-extreme value (58). Three data points were identified as extremely low at baseline and recoded. Given different units of measurement across modes of cannabis, the total amount of cannabis product used for each mode was first standardized using a z-score transformation. Z-scores were calculated separately for each group and time point. An average standardized score was calculated to reflect the overall amount of cannabis used across modes. Extreme outliers were recoded using the same method as for days of cannabis use. For quantity, 42 data points were identified as extremely high outliers and recoded.

For the three-month control group comparison of frequency of cannabis use, a one-way analysis of covariance (ANCOVA; three groups) was conducted contrasting the WB and WMI groups with the DT group, covarying the pretreatment value. The variable reflecting the overall quantity of cannabis use was highly skewed. Therefore, we ran a quade non-parametric ANCOVA (59); 3 groups], covarying the pretreatment value. Quade’s ANCOVA tests the equality of the residuals among groups using ranked covariates and the response variable (60). Additionally, to examine clinical significance, we compared a categorization between groups of the percentage of participants abstinent, improved (50% or greater reduction in days of cannabis use), and not improved (Hodgins et al. (28) using a monte carlo chi-square simulation with 10,000 replications given several low cell sizes.

To conduct the hypothesized comparisons of groups over the 6-month follow-up period, generalized equation estimations (GEE) were used for separate days of cannabis use and quantity of cannabis used, with participants as the subject variable, group as a fixed factor, time (0, 3, 6) as a fixed covariate, and assuming an AR1 correlation structure. Those who had a baseline assessment without completing follow-up assessments contributed only baseline data to the GEE model estimates. The slopes representing improvement from baseline to three months were expected to be larger than the slope from 3 to 6 months. Therefore, we modeled these slopes using a piece-wise linear approach. The DT group was coded as the reference condition.

GEE analyses also compared groups at 3-months and 6-months follow-up on secondary outcome variables: cannabis-related problems, psychological distress, and quality of life. Self-rated improvement across groups was compared using one-way ANOVA.

## Results

### Participation flow

**FIGURE 1:**
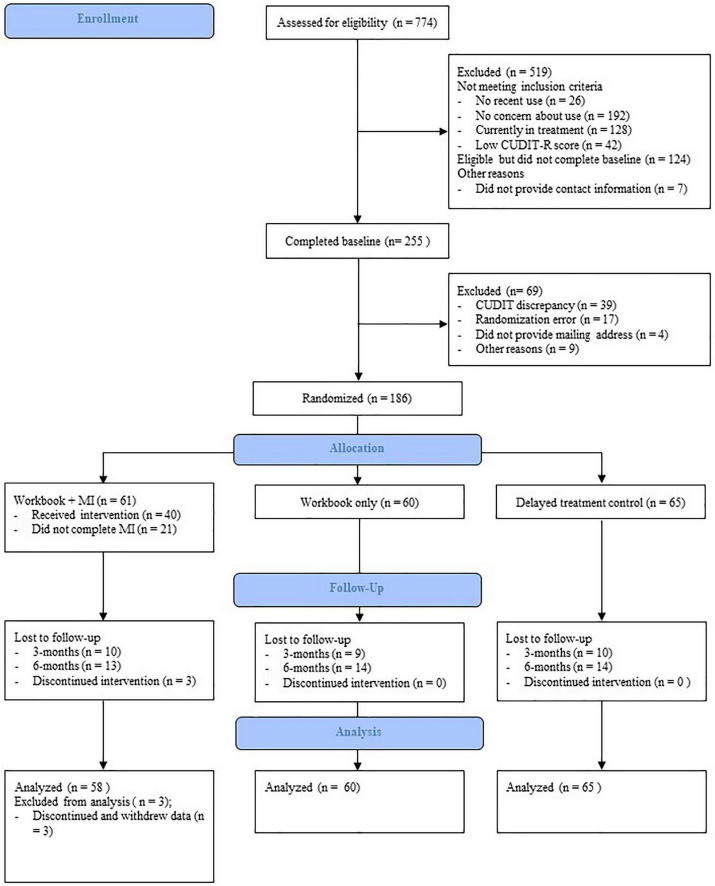
Consolidated Standards of Reporting Trials (CONSORT) style flow-chart of participant recruitment and retention.

Figure 1 provides the Consolidated Standards of Reporting Trials (CONSORT) style flow-chart of participants. Between December 2020 and April 2021, a total of 774 people were recruited, of whom 405 (52.33%) met initial eligibility criteria. Of the eligible participants, 255 (62.96%) completed the baseline survey. A further 69 participants were excluded from the study. See Figure 1 for a list of reasons for exclusion. “Other” reasons were idiosyncratic and impacted the ability to contact participants, such as emails not able to be delivered or a US mailing address. The remaining participants (*n* = 186; 72.94%) were randomly assigned to one of three groups, stratified by gender and problem severity. Of the 186 participants enrolled and randomly assigned to a condition, three discontinued and withdrew their data.

### Baseline characteristics

The final sample consisted of 183 participants (86 male; 46.99%; Table 2), aged 18 to 60 (*M* = 30.85; SD = 9.67), mostly Caucasian (*n* = 151; 82.51%), single (*n* = 112; 61.20%), and employed (*n* = 144; 78.69%).

**TABLE 2:** Participant demographic characteristics by group and across entire sample.

Characteristic, *n* (%)	Workbook + MI (*n* = 58)	Workbook only (*n* = 60)	Delayed workbook treatment (*n* = 65)	Total (*N* = 183)
Age, *M* (SD) *years*	30.48 (9.26)	31.32 (10.70)	30.74 (9.15)	30.85 (9.67)
Gender				
Female	28 (48.28)	28 (46.67)	30 (46.15)	86 (46.99)
Male	27 (46.55)	29 (48.33)	30 (46.15)	86 (46.99)
Non-binary	2 (3.45)	3 (5.00)	3 (4.62)	8 (4.37)
Other	1 (1.72)	–	2 (3.08)	3 (1.64)
Marital status				
Single (not legally married)	35 (60.34)	40 (66.67)	37 (56.92)	112 (61.20)
Legally married	6 (10.34)	7 (11.67)	7 (10.77)	20 (10.93)
Common-law	12 (20.69)	11 (18.33)	18 (27.69)	41 (22.40)
Separated	3 (5.17)	1 (1.67)	–	4 (2.19)
Divorced	2 (3.45)	1 (1.67)	3 (4.62)	6 (3.28)
Education				
High school or less	21 (36.31)	28 (46.67)	17 (26.15)	66 (36.07)
Trades or apprenticeship	1 (1.72)	3 (5.00)	–	4 (2.19)
College certificate/diploma	11 (19.97)	9 (15.00)	15 (23.08)	35 (19.13)
Some university	5 (8.62)	5 (8.33)	6 (9.23)	16 (8.74)
Undergraduate degree	12 (20.69)	9 (15.00)	16 (24.52)	37 (20.22)
Graduate degree	8 (13.79)	6 (10.00)	11 (16.92)	25 (13.66)
Employment^a^				
Full-time	22 (37.93)	28 (46.67)	30 (46.15)	103 (56.28)
Part-time	16 (27.59)	10 (16.67)	15 (23.08)	41 (22.40)
Unemployed	8 (13.79)	15 (25.00)	12 (18.46)	29 (15.85)
Retired	1 (1.72)	1 (1.67)	–	2 (1.09)
Student	15 (25.86)	13 (21.67)	17 (26.15)	45 (24.59)
Other	6 (10.34)	3 (5.00)	3 (4.62)	12 (6.56)
Income				
Under $10,000	6 (10.34)	6 (10.00)	3 (4.62)	15 (8.20)
$10,000 to $39,999	22 (37.93)	22 (36.67)	26 (40.00)	70 (38.35)
$40,000 to $69,999	13 (22.41)	13 (21.67)	16 (24.62)	42 (22.95)
$70,000 to $99,999	10 (17.24)	10 (16.67)	9 (13.85)	29 (15.85)
Over $100,000	7 (12.07)	9 (15.00)	11 (16.92)	27 (14.75)
Ethnicity^a^				
Caucasian	47 (81.03)	46 (76.67)	58 (89.23)	151 (82.51)
South Asian	2 (3.45)	3 (5.00)	4 (6.15)	9 (4.92)
Black	1 (1.72)	1 (1.67)	2 (3.08)	4 (2.19)
Latin American	3 (5.17)	2 (3.33)	–	5 (2.73)
Indigenous	2 (3.45)	5 (8.33)	2 (3.08)	9 (4.92)
Other	6 (10.34)	4 (6.67)	1 (1.54)	11 (6.01)

^a^participants could endorse multiple options.

Cannabis engagement characteristics at baseline are reported in Table 3. Participants reported using cannabis an average of 26.09 days in the past month (SD = 6.46). A majority of participants had a history of at least one previous attempt to reduce their cannabis use (*n* = 161; 87.98%), but few had ever sought treatment (*n* = 31; 16.94%). Most participants were interested in reducing their use versus stopping completely (*n* = 37; 20.22%).

**TABLE 3:** Cannabis engagement characteristics and scores on external measures at baseline across entire sample.

Characteristic, *M* (SD)	Workbook + MI (*n* = 58)	Workbook only (*n* = 60)	Delayed workbook treatment (*n* = 65)	Total (*N* = 183)
Use - Dried Cannabis Product, *n (%)*	56 (96.55)	59 (98.33)	64 (98.46)	179 (97.81)
Frequency (days)	24.13 (8.89)	24.08 (8.56)	23.48 (8.62)	23.88 (8.64)
Daily sessions	3.12 (1.63)	3.09 (1.98)	2.94 (1.75)	3.04 (1.79)
Daily product (grams)	3.70 (8.47)	2.11 (2.16)	2.51 (4.22)	2.75 (5.52)
Average THC (%)	20.63 (8.62)	19.57 (3.38)	20.42 (5.68)	20.20 (6.18)
Use-Concentrated cannabis products, *n (%)*	36 (62.07)	27 (45.00)	33 (50.77)	96 (52.46)
Frequency (days)	13.75 (11.36)	9.56 (9.98)	9.85 (9.55)	11.23 (10.46)
Daily sessions	2.83 (2.50)	4.67 (9.32)	2.48 (1.91)	3.32 (5.30)
Daily product (hits)	24.67 (43.68)	23.73 (48.08)	14.81 (19.30)	21.05 (38.52)
Average THC (%)	58.33 (26.66)	59.04 (22.62)	63.47 (20.21)	60.28 (23.33)
Use-Edible products, *n (%)*	30 (51.72)	36 (60.00)	45 (69.23)	111 (60.66)
Frequency (days)	7.60 (8.87)	5.39 (5.61)	5.56 (5.07)	6.05 (6.47)
Daily sessions	2.13 (1.74)	1.50 (0.94)	1.53 (0.99)	1.68 (1.24)
Daily product (grams)	19.32 (43.21)	7.21 (10.70)	12.22 (17.45)	12.27 (24.09)
Average THC per session (mg)	155.34 (237.72)	77.37 (113.47)	104.97 (204.96)	110.59 (192.61)
Frequency of overall cannabis use (days)	25.38 (7.17)	26.11 (6.59)	26.72 (5.65)	26.09 (6.46)
Age of first use	15.71 (2.58)	16.24 (3.67)	16.51 (3.71)	16.16 (3.38)
Age of regular use	19.79 (4.85)	20.93 (8.66)	20.43 (7.26)	20.39 (7.10)
Years of regular use	10.26 (9.08)	9.31 (9.58)	9.38 (9.93)	9.64 (9.51)
History of reduce attempts, *n (%)*	50 (86.21)	56 (93.33)	55 (84.62)	161 (87.98)
History of treatment seeking, *n (%)*	16 (27.59)	6 (10.00)	9 (13.85)	31 (16.94)
Abstinence goal, *n (%)*	11 (18.97)	12 (20.00)	14 (21.54)	37 (20.22)
CUDIT-R	22.24 (4.29)	22.53 (4.52)	22.26 (4.45)	22.34 (4.40)
MPS	12.83 (6.86)	12.88 (7.28)	11.95 (6.21)	12.54 (6.76)
MPS-L	13.84 (6.77)	12.47 (6.46)	12.94 (6.80)	13.07 (6.66)
K10	29.81 (7.98)	27.48 (8.72)	27.80 (8.75)	28.33 (8.52)
WHOQoL-8	3.08 (0.72)	3.14 (0.84)	2.94 (0.77)	3.05 (0.78)

CUDIT-R, cannabis use disorders identification test-revised; MPS, marijuana problem scale; MPS-L, marijuana problem scale-lifetime version; K10, kessler psychological distress scale; WHOQoL-8, world health organization quality of life-8 item scale.

Regarding engagement in other potentially addictive substances and behaviors, participants showed the highest scores on the SSBA subscales of cannabis (M = 9.66, SD = 3.90) tobacco (M = 7.15, SD = 5.06), and eating (M = 5.87, SD = 4.07). The average SSBA subscale scores across each group and the entire sample are shown Supplementary Table 1.

Among participants who were randomly assigned to the condition which included a motivational telephone interaction, those who completed and did not complete the interview were compared on the variables displayed in Tables 2, 3, and Supplementary Table 1. No significant differences emerged on any demographic characteristics or SSBA subscale scores, *p*s > 0.06. Not assuming equal variances, individuals who did not complete the MI interview reported greater THC in the concentrated cannabis products used compared to individuals who completed the interview, *t*(31.49) = −2.41, *p* = 0.02, and a higher frequency of cannabis use across modes, *t*(55.90) = 2.30, *p* = 0.03, than individuals who completed the interview.

Participants who did and did not complete the follow-up assessments at 3 and 6 months were also compared on the same variables. Individuals with fewer daily reported sessions of dry cannabis use were less likely to complete the 3-month follow-up, *t*(176) = −1.98, *p* = 0.049. Individuals with higher scores on the MPS-L were also less likely to have completed the follow-up at three months, *t*(178) = 2.40, *p* = 0.01, and at 6 months, *t*(178) = 1.81, *p* = 0.04. Not assuming equal variances, individuals who did not complete the 6-month follow-up also reported significantly greater THC in their concentrated cannabis products, *t*(60.41) = 2.93, *p* = 0.002, but less THC in edibles, *t*(75.88) = −2.44, *p* = 0.01.

Finally, participants were asked whether they had sought other professional treatment during the follow-up window. The overall proportion of participants seeking other professional support was 18.46% at 3-months and 22.46% at 6-months, with no between-group differences, *p*s = 0.84 and.43. ANCOVA revealed that seeking other professional support did not predict the frequency of cannabis use at 3-months, *F*(1, 144) = 3.12, *p* = 0.08, or at 6-months, *F*(1, 130) = 0.08, *p* = 0.77. Neither did it predict the proportion of cases improved or abstinent at 3-months, χ^2^(2) = 5.84, *p* = 0.08, or 6-months, *p* = *0.81*.

### Group comparisons at 3-months

Results partially supported our first hypothesis that participants in the WMI and WB groups would show lower frequency of cannabis use at 3-months compared to the DT group. For days of cannabis use, an ANCOVA was conducted, covarying the days of cannabis use in the month prior to beginning the study^1^. Although the assumption of homogeneity of variances was not met, Levene’s *F*(2, 148) = 3.73, *p* = *0.03*, the F-test is robust to the variance ratio and coefficient of sample size variation observed (61). Therefore, it was appropriate to move forward with the untransformed data. There was a statistically significant difference in days of cannabis use between the groups, *F*(1, 2) = 5.16, *p* = 0.007, partial η^2^ = 0.07. Controlling for baseline days of cannabis use, frequency of use at three months was significantly lower WMI group (*M* = 16.83, SD = 11.97) versus the DT group (*M* = 22.67, SD = 9.55), mean difference of −3.83, 95% CI [−7.27, −0.38], *p* = 0.03. However, days of cannabis use did not differ significantly between the WB (*M* = 22.76, SD = 8.99) and DT group, mean difference = 1.75, 95% CI [−1.64, 5.15], *p* = 0.31. Results also supported our third hypothesis that participants in the WMI group would show a greater reduction than participants in the WB group, mean difference = −5.58, 95% CI [−9.08; −2.08].

The proportion of participants abstinent, improved (50% or greater reduction in days of cannabis use), and not improved are shown in Table 4. Monte carlo simulation analyses showed a statistically significant association between group and outcomes χ^2^(4) = 9.52, *p* = 0.046; a greater number of individuals in the WMI group improved or achieved abstinence compared to the other two groups at *p* < 0.05.

**TABLE 4:** Classification of outcome based on days of cannabis use *n* (%).

Follow-up	Workbook + MI	Workbook only	Waitlist control
3 months	*n* = 47	*n* = 47	*n* = 55
Abstinent	5 (10.64)	1 (2.13)	1 (1.82)
Improved	12 (25.53)	6 (12.77)	8 (14.55)
Not improved	30 (62.83)	40 (85.11)	46 (83.64)
6 months	*n* = 45	*n* = 43	*n* = 48
Abstinent	4 (8.89)	3 (6.98)	1 (2.08)
Improved	11 (24.44)	10 (23.26)	8 (16.67)
Not improved	29 (64.44)	30 (69.77)	39 (81.25)

Participants quantities of cannabis used were compared with Quade’s ANCOVA, covarying the baseline quantity. There was a statistically significant difference in quantity of cannabis used between groups, Quade’s *F*(2, 147) = 3.12, *p* = 0.047. Consistent with hypothesis 3, quantity of cannabis use was significantly lower in the WMI group compared to the WB group, *t*(147) = −2.37, *p* = 0.02). When the analysis was rerun without recoding the extreme outliers, the significant effect was no longer present.

### Group comparisons over six months^2^

**FIGURE 2:**
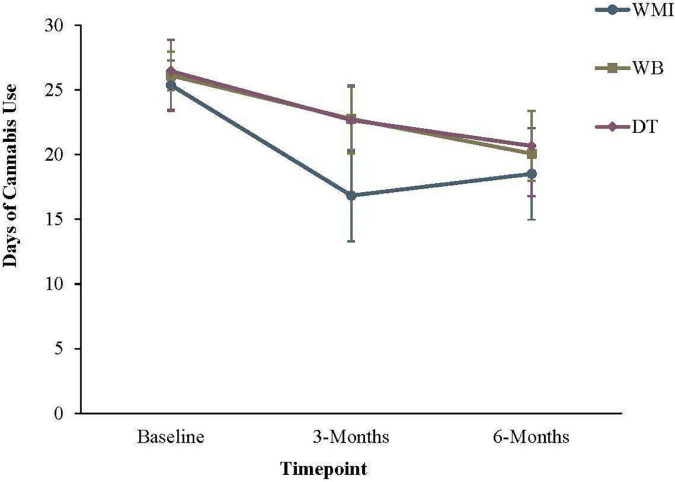
Frequency of cannabis use in the previous 30 days. Error bars represent the 95% confidence interval for that group at a given timepoint. WMI, workbook plus motivational interview group; WB = workbook only group; DT = delayed workbook treatment group.

The groups means at baseline and the two follow-up periods are displayed in Table 5. For days of cannabis use, GEE modeling revealed that the baseline to 3-month slopes differed significantly from zero, *ps* < 0.001. Results also partially supported our second hypothesis; the baseline to 3-month slope for the WMI group differed significantly from the DT group (see Table 6 and Figure 2). However, the slope for the WB group did not differ significantly from the DT condition. Consistent with hypothesis 2, when the WMI group was contrasted against the WB group, a significant effect emerged. Between baseline and 3-months, individuals in the WMI condition showed a significantly greater reduction in days of cannabis use than individuals in the WB alone group, *Est(SE)* = −5.23(1.84), Wald = 8.09, *p* = 0.005. The 0- to 6-month slope was significant, *Est(SE)* = −5.99(1.17), Wald = 26.39, *p* < 0.001. However, the 3-to 6-month slope only approached significance, *p* = 0.06. The 3- to 6-month slope for the WMI group continued to differ significantly from the DT group, but in the opposite predicted direction, *Est(SE)* = 4.62(2.11), Wald = 4.78, *p* = 0.03. At 6 months, the means for the DT group did not differ significantly from the WB group, *p* = 0.90.

**TABLE 5:** Means (and SDs) for primary and secondary outcomes at baseline and follow-up assessments.

	Baseline	3 months	6 months
Days			
Workbook + MI	25.38 (7.17)	16.83 (11.97)	18.35 (11.73)
Workbook only	26.11 (6.59)	22.76 (8.99)	20.07 (10.93)
Delayed workbook treatment	26.72 (5.65)	22.67 (9.55)	20.67 (9.37)
Amount^a^			
Workbook + MI	0.08 (0.67)	−0.12 (0.10)	−0.08 (0.20)
Workbook only	0.04 (0.94)	−0.08 (0.16)	−0.08 (0.19)
Delayed workbook treatment	−0.12 (0.18)	−0.11 (0.08)	−0.08 (0.18)
MPS			
Workbook + MI	12.83 (6.86)	8.72 (7.77)	8.91 (7.52)
Workbook only	12.88 (7.28)	10.23 (6.40)	9.73 (8.19)
Delayed workbook treatment	11.95 (6.21)	9.41 (6.57)	8.50 (5.69)
K10			
Workbook + MI	29.81 (7.98)	25.43 (8.17)	25.56 (8.10)
Workbook only	27.48 (8.72)	24.60 (8.33)	23.98 (7.34)
Delayed workbook treatment	27.80 (8.75)	25.13 (8.68)	25.02 (8.42)
WHO QoL			
Workbook + MI	3.08 (0.72)	2.45 (0.68)	2.70 (0.75)
Workbook only	3.14 (0.84)	2.38 (0.86)	2.64 (0.81)
Delayed workbook treatment	2.94 (0.77)	3.19 (0.77)	2.81 (0.84)
Self-Rated Improvement			
Workbook + MI	–	5.24 (3.02)	4.51 (2.86)
Workbook only	–	4.00 (2.00)	4.33 (2.80)
Delayed workbook treatment	–	3.30 (2.71)	4.18 (2.32)

^a^Z-score transformed variable. MPS, marijuana problem scale; K10, kessler psychological distress scale; WHOQoL-8, world health organization quality of life-8 item scale.

**TABLE 6:** Parameter estimates for days and quantity of cannabis use from GEE modeling.

Effect	Parameter estimate	SE	Wald	*P*	95% CI
Days of cannabis use					
DT Intercept	26.43	0.75	1253.87	< 0.001	24.96, 27.90
WMI	–1.10	1.19	0.86	0.35	−3.45, 1.24
WB	–0.69	1.69	0.34	0.56	−2.98, 1.62
Baseline to 3-month slope					
DT	–3.94	1.11	12.53	< 0.001	−6.14, −1.75
WMI	–4.21	1.82	5.34	0.02	−7.79, −0.63
WB	1.02	1.59	0.41	0.52	−2.12, 4.15
3-month to 6-month slope					
DT	–2.42	1.32	2.37	0.07	−5.02, 0.18
WMI	4.62	2.11	4.78	0.03	0.46, 8.79
WB	–0.23	1.78	0.02	0.90	−3.73, 3.28
Quantity of cannabis use (Composite)					
DT Intercept	–0.07	0.06	1.34	0.25	−0.18, 0.05
WMI	–0.09	0.06	1.96	0.16	−0.21, 0.04
WB	–0.11	0.06	3.42	0.06	−0.22, 0.01
Baseline to 3-month slope					
DT	0.06	0.09	0.42	0.52	−0.11, 0.23
WMI	0.02	0.11	0.05	0.83	−0.20, 0.24
WB	0.14	0.13	1.10	0.30	−0.12, 0.389
3-month to 6-month slope					
DT	–0.12	0.07	2.62	0.06	−0.25, 0.005
WMI	0.04	0.09	0.18	0.68	−0.15, 0.23
WB	–0.09	0.11	0.69	0.41	−0.30, 0.12

The delayed workbook treatment (DT) group is the reference condition to which the workbook plus MI (WMI) and the workbook only (WB) groups are compared CI, confidence interval.

**FIGURE 3:**
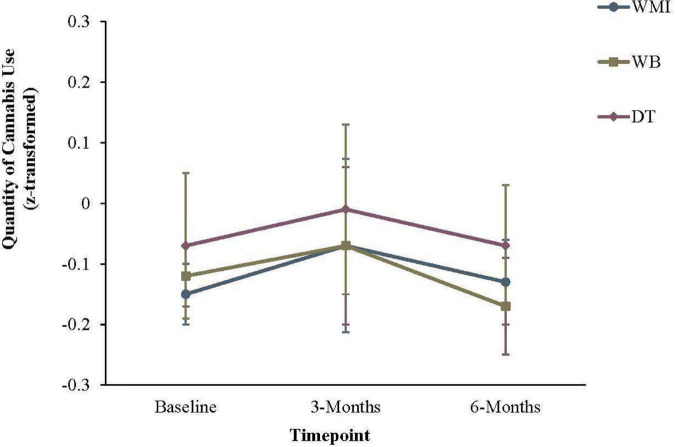
Quantity of cannabis use in the previous 30 days. Error bars represent the 95% confidence interval for that group at a given timepoint. WMI, workbook plus motivational interview group; WB, workbook only group; DT, delayed workbook treatment group.

For quantity of cannabis use, a significant effect of time emerged, χ^2^(2) = 12.20, *p* = 0.002 (Table 6 and Figure 3). However, no between-group effects were significant from baseline to 3-months, or from 3- to 6-months, *p*s > 0.06.

### Secondary outcomes

GEE modeling compared groups on problems associated with cannabis use (MPS), psychological distress (K10) and quality of life (WHOQoL-8). Across outcomes, a significant effect of time emerged, *ps* < 0.001. However, the groups did not differ significantly across time from one another, *p*s > 0.13.

Participants were asked at each follow-up how successful they felt they had been at reaching their treatment goal in the preceding 3-months. A one-way ANOVA compared participants self-rated success at each follow-up. Self-rated success differed significantly between groups at 3-months, *F*(2, 145) = 6.02, *p* = 0.003, but not at 6-months, *p* = 0.90. *Post hoc* analyses with the holm-bonferroni adjustment indicated that the participants in the WMI group had significantly higher self-rated success (*M* = 5.24, SD = 3.02) at 3-months than both the DT (*M* = 3.43, SD = 2.83) and WB (*M* = 4.00, SD = 1.98) groups, *p*s.048 and <0.001. The difference between the WB and DT groups was not significant, *p* = 0.82. No significant between-group differences emerged on the perceived helpfulness of the workbook or how often the workbook was used at either 3-months or 6-months, *p*s > 0.14. A follow-up linear regression analysis examined whether the frequency of workbook use predicted cannabis use at 3- and 6-months follow up. Controlling for the frequency of cannabis use at baseline, workbook use did not predict cannabis use at 3-months follow-up, *p* = 0.09. However, frequency of the workbook between 3- and 6-months predicted days of cannabis use at 6-months follow-up, *B* = −3.16, SE = 1.03, *t* = −3.06, *p* = 0.003.

## Discussion

Overall, the primary hypotheses were partially supported. The workbook in combination with a motivational interview (WMI) demonstrated its utility at reducing the frequency of cannabis use compared to both the workbook (WB) and delayed workbook treatment (DT) condition. Individuals in the WMI condition reported significantly fewer days of cannabis use at 3-months follow-up compared to those who received the workbook alone (WB) or in the delayed workbook treatment group (DT), lending support for hypotheses 1 and 3. When considering the number of participants who had improved or achieved abstinence across the first three-months, a similar pattern emerged; Individuals in the WMI group showed significantly greater rates of abstinence compared to the other groups than would be expected by chance.

Between baseline and 3-months, individuals in the WMI condition showed a significantly greater reduction in days of cannabis use than individuals in the WB alone group. The 3-to 6-month slope for the DT group only approached significance, indicating that continued improvement slowed after 3-months. This is not surprising, given the level of improvement observed in this group before receipt of the workbook. Surprisingly, the 3- to 6-month slope for the WMI group continued to differ significantly from the DT group, but in the opposite predicted direction, indicating that use rose slightly between 3- and 6-months. Future research might consider whether a booster MI session would help sustain the changes made in the first three months. Walker and colleagues (62) previously found that MI maintenance check-ups at 1- and 4-months post-treatment led to greater rates of abstinence than participants who did not receive subsequent MI sessions following a CBT/MET treatment for CUD. It is possible that additional MI as needed could help sustain the greater rate of change that was seen in the WMI group between 0- and 3-months.

For quantity of cannabis use, a significant effect of time emerged, but no between-group effects were significant from baseline to 3-months, or from 3- to 6-months. This was somewhat surprising, given the changes in the frequency of cannabis use that was observed in the current study. One possible explanation for the effect is that individuals may have initially increased the quantity of their cannabis use while attempting to reduce the overall frequency. Indeed, Figure 3 shows a small increase in overall quantity of use between baseline and 3 months, before a decrease between 3- and 6-months. However, we cannot conclude whether or not this effect was simply due to chance, as none of the results were statistically significant. We were also required to z-score transform the measures of quantity, which would have reduced variability and possibly reduced the power to statistically detect changes in quantity of cannabis.

All groups showed similar rates of improvement in self-reported quality of life, reduced psychological distress, and fewer problems associated with their cannabis use through the course of the study. It is unclear whether this is due to the changes in the frequency and quantity of cannabis use that was observed across groups as well. Similarly, no significant between-group differences emerged on the perceived helpfulness of the workbook or how often the workbook was used at either 3-months or 6-months. However, linear regression analyses revealed that while frequency of workbook use in the first three months did not predict frequency of days of cannabis use at the 3 month-follow-up, use between 3- and 6-months predicted days of cannabis use at the 6-month follow-up. This suggests that while continued improvement slowed after the first 3-months, higher use of the workbook predicted lower rates of cannabis use at 6-months follow-up. This finding lends some support to the clinical utility of the workbook itself.

To our knowledge, this is the first study to examine the utility of a self-directed treatment workbook for problematic cannabis use as opposed to web-based treatment programs (32, 33). In contrast to the study by Rooke et al. (32) and our own hypotheses, we found no difference between the workbook alone and the control group on frequency of cannabis use at 3-months follow-up. In our study, the frequency of cannabis use decreased across participants, including those in the DT condition. However, previous research has also shown that problematic cannabis use can change over time, without formal intervention (25). Participants in the current study were motivated to reduce their cannabis use and many had attempted to change their cannabis in the past. Additionally, some participants, including those in the DT condition, sought other supports through the duration of this study, demonstrating a continuing desire to change their cannabis use. It is also possible that completion of the baseline assessment heightened participants’ awareness of their current problems and thus increased their motivation to change. The baseline assessment included questions designed to assess problems associated with cannabis use, severity, and frequency. These areas are also explored in brief interventions, which aim to increase awareness and motivation for change (63, 64). Similar strategies and tools are also included in the workbook to support self-assessment and reflection. Thus, the need to include a detailed baseline assessment may have confounded the benefit of the workbook.

Several limitations of this study should be noted. First, because the delayed treatment period was limited to 3-months, it is not possible to examine the efficacy of the intervention for longer follow-up to a no-intervention group. As noted, all participants reduced their cannabis use in the first three months, at which point DT participants then received the workbook. It is possible that access to the workbook contributed to the continued changes between 3- and 6-months, whereas without access, rate of change would have slowed to a greater extent. As noted, using the workbook more frequently predicted lower rates of cannabis use at 6-months follow up. A second limitation is that quantity of cannabis use was measured by averaging z-score transformed measures of quantity across the three modes of cannabis use. This inherently creates challenges with interpretability and possibly limited our ability to detect between-group differences. Unfortunately, the field lacks a standardized method of assessing quantity of cannabis consumption across various modes. As previously described (51), participants struggle to estimate the amount of concentrated cannabis products used and so the CEA asks participants to report the number of “hits” rather than milligrams of cannabis itself. The most commonly used concentrate product is oil for vaping (51), where CBD and THC are suspended in an oil solution with varying density. This makes it impossible to calculate the amount of cannabis itself consumed in each hit. Thus, we can estimate the amount of product used, though not the amount of cannabis. Nevertheless, with the composite variable, we were able to track changes in cannabis consumption over time and across multiple modes of use. Other studies, such as Rooke and colleagues (32) did not assess cannabis use across the myriad ways in which it can be consumed. A third related limitation is that assessing the frequency of cannabis use is also an imperfect outcome variable, given that some people sought to achieve abstinence rather than reduce their cannabis use. Fourth, we were unable to explore the effects of the intervention on THC quantity, or the influence of THC quantity on the results. Participants inconsistently reported their THC usage, with a majority not able to provide an estimate. As such, THC quantity was an unreliable index of use. Fifth, we estimated the sample size needed to detect a reduction of 10 days or more of cannabis use, though the intervention did not lead to a reduction of that amount. Thus, the study may have been underpowered. However, the results from this study can inform the target sample size of future research. Sixth, participants self-reported their cannabis use and we did not include an objective measure or collateral reports. However, it was not feasible to collect more objective measures in the current study, as we recruited participants from across Canada. Additionally, the sample included participants who were interested in a low-intensity treatment. Collection of urinalysis or saliva would have changed the representativeness of the sample and may have greatly increased attrition rates. Previous research has found high rates of concordance between urinalysis or collateral information and rates of abstinence [e.g., (62)]. This strengthens confidence in the validity of the self-reported cannabis outcomes.

## Conclusion and future directions

The current study highlighted the utility of a brief motivational interview in combination with a self-directed workbook at promoting changes in cannabis use. Given many individuals with problematic cannabis use do not seek formal treatment (21, 22), this self-directed intervention has the potential to fill an important need in that the self-directed intervention can attract individuals who wish to recover with minimal professional support.

Individuals who use cannabis are also diverse in terms of both demographic factors and treatment goals and needs. However, many treatment programs emphasize only abstinence as a recovery outcome (9), rather than also supporting moderated use. This may partly explain why treatment-assisted participants with moderation goals are less likely to recommend treatment-assisted recovery (39). As such, a range of intervention possibilities of varying intensities, and the ability to personalize treatment goals is ideal (5). A stepped-care approach may enhance the provision of treatment for CUD by providing individuals with the opportunity to choose a level of intervention and treatment that is consistent with their goals and preferences. The workbook package tested in this study is sensitive to individual treatment goals, whether abstinence or controlled use. In fact, most participants were interested in reducing their use rather than stopping completely.

We have identified several avenues of future research. First, it would be beneficial to consider the efficacy of the intervention in a larger sample and over a longer follow-up period. As noted, an additional booster MI phone call could help sustain the changes made in the first three months and consequently, this should be investigated. Second, while stepped-care models consider many elements of an integrated public health response to preventing and treating problematic cannabis use, little research has sought to integrate such elements. Future research may benefit from examining uptake of our self-directed intervention through other resources within a stepped-care framework. For example, we previously proposed that this intervention could be integrated with Screening, Self-Management and Referral to Treatment (SSMRT), a secondary prevention platform designed to reduce harms from cannabis use, provide information, and connect interested individuals to appropriate treatments (65). Third, future research would also benefit from increasing our understanding of individual differences in treatment responsiveness among individuals with various demographic and treatment goals, and who are interested in self-directed change. Such information could inform refinement of treatment resources that are sensitive to the experiences and needs of individuals with cannabis problems. Relatedly, CUD is commonly comorbid with other mental health concerns (66). Future research may also consider the influence of comorbid mental health conditions on responsiveness to self-directed treatment. This line of research would also shed further light on important considerations for a successful public health approach.

## Data availability statement

The raw data supporting the conclusions of this article will be made available by the authors, without undue reservation.

## Ethics statement

The studies involving human participants were reviewed and approved by University of Calgary Conjoint Faculties Research Ethics Board (CFREB). The patients/participants provided their written informed consent to participate in this study.

## Author contributions

MS and DH contributed to the conceptualization and design of the study. JS and MK critically reviewed the design and grant proposal. MS performed the statistical analyses and drafted the first manuscript. DH wrote parts of the manuscript and edited subsequent versions. JS and MK reviewed the manuscript for critical content. All authors contributed to manuscript revision, read, and approved the submitted version.

## Footnotes

1 The analysis was rerun without recoding the extreme outliers and yielded similar results.

2 GEE models were rerun without recoding the extreme outliers and yielded similar results.
