# Supplementary material for: Promoting self-change in cannabis use disorder: Findings from a randomized trial
Source: Front Psychiatry. 2022 Nov 22;13:1015443. doi: 10.3389/fpsyt.2022.1015443 (PMC9722728; doi:10.3389/fpsyt.2022.1015443)
Supplement: Supplementary file 1 [file Table_1.DOCX]

**Table S1.** Participant scores on the Screener for Substance and Behavioural Addictions (SSBA) sub-scales for each group and across the whole sample.

| Sub-Scale | WMI | WB | WC | Overall Sample |
| --- | --- | --- | --- | --- |
| Alcohol | 3.47 (3.74) | 3.66 (4.30) | 3.51 (3.80) | 3.54 (3.90) |
| Tobacco | 6.33 (5.66) | 8.79 (5.13) | 6.66 (4.37) | 7.15 (5.06) |
| Cannabis | 10.20 (3.75) | 9.53 (4.17) | 9.28 (3.78) | 9.66 (3.90) |
| Cocaine | 5.78 (5.49) | 2.86 (3.34) | 1.77 (1.69) | 3.28 (3.92) |
| Gambling | 2.75 (2.45) | 2.28 (3.21) | 3.36 (3.14) | 2.71 (2.95) |
| Shopping | 2.75 (2.86) | 2.34 (3.17) | 4.45 (4.25) | 3.51 (3.54) |
| Gaming | 4.04 (3.16) | 4.07 (4.26) | 2.07 (2.42) | 3.37 (3.46) |
| Eating | 4.97 (3.89) | 6.00 (4.54) | 6.44 (3.72) | 5.87 (4.07) |
| Sexual Activity | 4.03 (3.85) | 3.62 (4.00) | 3.21 (3.57) | 3.59 (3.79) |
| Working | 4.82 (3.85) | 2.44 (3.56) | 3.74 (4.08) | 3.63 (3.92)* |

* between-group difference significant at *p* < .05
